# Inequality gaps in modern contraceptive use and associated factors among women of reproductive age in Nigeria between 2003 and 2018

**DOI:** 10.1186/s12905-024-03167-z

**Published:** 2024-06-01

**Authors:** Obasanjo Afolabi Bolarinwa

**Affiliations:** 1 Department of Public Health, York St John University, London, UK; 2 Department of Demography and Population Studies, University of Witwatersrand, Johannesburg, South Africa

**Keywords:** Inequality, Modern contraceptives, WHO, HEAT, Women of reproductive age, Nigeria, DHS

## Abstract

**Background:**

Inequalities in modern contraceptive use among women in low-income countries remain a major public health challenge. Eliminating or reducing the inequalities in modern contraceptive use among women could accelerate the achievement of Sustainable Development Goals, Targets 3.7 & 5.6. Thus, this study examined the inequality gaps in modern contraceptive use and associated factors among women of reproductive age in Nigeria between 2003 and 2018.

**Methods:**

This study employed the World Health Organisation’s Health Equity Assessment Toolkit to analyse the 2003 and 2018 Nigeria Demographic Health Surveys. Modern contraceptive use was aggregated using five equity stratifiers: age, economic status, educational level, place, and region of residence among women of reproductive aged 15 to 49, with a sample size of 5,336 and 29,090 for 2003 and 2018, respectively. Inequality was measured in this study using difference (D), ratio (R), population-attributable risk (PAR), and a population-attributable fraction (PAF).

**Results:**

The study shows an increase in modern contraceptive use among women of reproductive age in Nigeria from 8.25% in 2003 to 12.01% in 2018, with the use being more prominent among women of reproductive age 20–49 and those in the richest economic quintile. In both surveys, women with primary education showed the most upward increase in modern contraceptive use. Women residing in the urban areas also show an upward use of modern contraceptives use. The study further highlights inequality gaps, with age being a substantial factor, while economic status and sub-national regions showed mild to marginal inequality gaps. Finally, the educational level of women of reproductive age in Nigeria significantly shows inequality in modern contraceptive use, with a PAF of 129.11 in 2003 and 65.39 in 2018.

**Conclusion:**

The inequality gap in modern contraceptive use among women of reproductive age in Nigeria between 2003 and 2018 reported in this study includes age, education, wealth quintile, residence, and region-related inequalities. The study highlights the need for policies and programmes that target the groups with low use of modern contraceptives to promote equity in family planning services.

## Background

In the year 2020, the United Nations Population Fund (UNFPA) reports showed that 58% of women of reproductive age globally were using modern contraceptives [1], categorising more than 40% of women in the same age group as non-users of modern contraceptives globally [1]. At the same time, outstanding progress in modern contraceptive use has also been recorded in low-and middle-income countries (LMICs) since its wide acceptance in the 20th century [2].

The achievement of this progress could be attributed to the benefits and convenience associated with using modern contraceptives [3, 4]. Even though the users of modern contraceptives among women globally is slightly above average, there is still a significant inequality gap in modern contraceptive use between different regions and socioeconomic groups in sub-Saharan Africa (SSA), which influences the decision to use modern contraceptives or not [3, 5].

Moreover, some factors like regions of residence and socioeconomic status of women have been reported to show significant disparities in modern contraceptive use among women of reproductive age [3, 6]. For instance, in SSA, only 29% of women of reproductive age use modern contraceptives, compared to 70% in Eastern Asia [7]. In the same vein, women living in rural areas, those with lower income and education, are less likely to use modern contraceptives compared to their counterparts [6].

Nigeria is the most populous country in Africa and also one of the countries that have reported significant progress in the acceptance and use of modern contraceptives among women in Africa after Rwanda and South Africa over the past two decades [8, 9]. Despite the widespread acceptability and increase in modern contraceptive use, this use is not homogenous among women of reproductive age due to inequalities between the socio-economic group and regional differences [10, 11].

For instance, the prevalence of modern contraceptive use among women in SSA was 33% [12], and according to the Demographic Health Survey (DHS), the modern contraceptive use prevalence in South Africa and Rwanda was 66.7% and 56.1% for women of reproductive age respectively, whilst Nigeria modern contraceptive use prevalence was 12% among the same group of women [13–15]. Both South Africa and Rwanda’s modern contraceptive use was above the regional average, whilst Nigeria was below the regional average.

Furthermore, Nigeria has a high fertility rate and unequal age-specific fertility rate distribution in Africa [16], coupled with a total fertility rate of 5.3 per woman of reproductive age and 20% of unmet needs for modern contraceptives [17]. The country’s high fertility rate and unmet need for modern contraceptives have been associated with inequalities in modern contraceptive use [18, 19]. Nevertheless, studies have failed to address these inequalities in modern contraceptive use among regions and some socioeconomic status in Nigeria, which continue to contribute to the high fertility rate the country is experiencing [20–22].

The low use and inequality in modern contraceptives among women of reproductive age in Nigeria have several serious implications for maternal and child health, continuous economic development, and gender equality in the country [22, 23]. In the same vein, uncurbed inequality in modern contraceptive use can lead to undesired fertility associated with various adverse reproductive health outcomes such as maternal and infant mortality, limited women empowerment, and low participation in social and economic events [24, 25]. Unlike previous studies that focused on prevalence, spatial distribution and associated factors that influence modern contraceptive use among women of reproductive age in Nigeria [11, 23, 26, 27], this current study focused on unearthing the inequalities gaps associated with modern contraceptive use among women of reproductive age in Nigeria between 2003 and 2018, on having the holistic view and trend of the progress since the beginning of the millennium.

Understanding the disparities across different socio-economic groups and regions of residence in relation to low modern contraceptive use aligns with Sustainable Development Goal (SDG) Target 5.6, which aims to achieve universal access to sexual and reproductive health and rights whilst also contributing to SDG Target 3.7, which ensures the achievement of universal access to sexual and reproductive healthcare services [1, 28]. By prioritising efforts to address the inequalities in modern contraceptive use in the country, Nigeria can work towards achieving sustainable development and leaving no one behind in the journey towards achieving remarkable sexual and reproductive health rights and services [28, 29].

It’s pertinent to note that there is a research gap in the use of the Health Equality Assessment Toolkit (HEAT) developed by the World Health Organisation (WHO) to assess and examine the variations in regional and socioeconomic inequalities using complex summary measures across equity dimensions on the use of modern contraceptives among women of reproductive age in Nigeria using different survey years [30].

Therefore, there is a need to understand the associated factors that influence the inequalities gap in modern contraceptive use among women of reproductive age in Nigeria; this understanding is essential for policymakers and program implementers in designing and implementing specific effective interventions that can improve the use of modern contraceptives in Nigeria by reducing the inequality gap associated with modern contraceptive use. Thus, this study assessed the inequality gaps in modern contraceptive use and associated factors among women of reproductive age in Nigeria between 2003 and 2018 using Nigeria Demographic Health Survey (NDHS) datasets facilitated by the WHO HEAT inequality software application.

## Methods and materials

### Study design and data source

A secondary cross-sectional dataset from 2003 and 2018 NDHS was analysed. The Demographic Health Survey (DHS) data are collected in more than 85 LMICs at 5-year intervals, including Nigeria [31]. The 2003 data collection was used as the base data because it was the first conclusive dataset collected in Nigeria with consistent and completed data points, while 2018 is the latest available dataset. The study considered the 2003 and 2018 datasets in assessing the inequality gap in modern contraceptive use among women of reproductive age in Nigeria. DHS focus on collecting information on the health profile of children, men, and women [32]. Among the important health profile information often collected by DHS include family planning use, maternal, child and newborn health and sexual health. DHS considers the selection of enumeration areas (EAs) as the first sampling point, which includes both rural and urban areas in Nigeria; the complete sampling procedure for 2003 and 2018 was expressly explained here [17, 31, 32]. The interested population for this study was women aged 15 to 49, and a total sample size of 5,336 and 29,090 for 2003 and 2018 NDHS was drawn for this study’s analysis.

### Variable of interest

#### Outcome variable

This study’s outcome variable of interest was modern contraceptive use among women of reproductive age in Nigeria between 2003 and 2018. In this study context, modern contraceptive use was considered as any woman of reproductive age using any method of modern contraception, such as oral contraceptive pills, implants, injectables, intrauterine devices, and condoms for males and females [33]. Women who responded “yes” to modern contraceptive use in both survey years were coded as “1”, and those who responded otherwise were coded as “0”. Similar measures have been used in previous studies on modern contraceptive use among women in Nigeria [6, 26, 34].

### Explanatory variables

To assess the inequality gap in modern contraceptive use in Nigeria between 2003 and 2018, five stratified were used, and this includes age (15–19 years, 20–49 years), economic status measured by wealth index between quintiles 1 to 5, educational attainment (no education, primary, secondary and above), place of residence (rural and urban), and finally, region of residence or sub-national region which include North Central, North East, North West, South East, South-South, and South West measured. The DHS measures the wealth index by aggregating household items such as televisions, bicycles, materials used for house construction, sanitation facilities and type of water access into five wealth quintiles, namely: poorest, poorer, middle, richer, and richest, which represent the quintile 1 to 5. The wealth index was derived using the continuous composite measure.

### Statistical analysis

The 2019 updated HEAT version 3.1 software was used for all the analyses included in this study [30]. To measure the inequality gap in modern contraceptive use among women of reproductive age in Nigeria between 2003 and 2018, five equity stratifiers were employed. The inequality computation included four measures, including difference (D), population-attributable risk (PAR), population-attributable fraction (PAF), and ratio (R). D and R represent the unweighted measure, while PAR and PAF represent the complex weighted measures. Summary measures were considered in this study because the WHO mentioned that absolute and relative measures are important in generating required policy-driven findings [30, 35].

To calculate for D in economic status, women using modern contraceptives in the poorest group were subtracted from the richest group; in the same vein, for educational attainment, women with no formal education were subtracted from those with secondary education above, etc. The R was computed for variables with ordered responses such as education and wealth quintile as the difference between the most disadvantaged sub-group as the lowest quintile and uneducated and the most advantaged sub-group such as the richest quintile and secondary above education. PAR was derived by computing the difference between women who use modern contraceptives in the reference category and the overall average of prevalence of women using modern contraceptives. A zero PAF or PAR means no inequality, while a higher value indicates a relatively greater inequality. The variation of modern contraceptive use among women of reproductive age in Nigeria over the considered period in this study was explored by referring to the 95% confidence interval (CIs) for each survey year. Sample weights were applied using the HEAT software to account for over-and undersampling in the surveys.

## Results

### Trends in modern contraceptive use inequality among women of reproductive age in Nigeria between 2003 and 2018

The analyses indicated a rise in modern contraceptive use among women of reproductive age in Nigeria from 8.25% in 2003 to 12.01% in 2018, given an upward increase of 3.76% in modern contraceptive use among women of reproductive age in Nigeria (Table 1). The use of modern contraceptives was more pronounced among women of reproductive age between the age of 20–49 in both surveys, with corresponding 8.75% in 2003 to 12.70% in 2018. Regarding the economic status of women, Nigerian women within the quintile 5 (richest) dominated the progressive use of modern contraceptives from 20.53 to 22.23% in 2003 and 2018, respectively. The educational level inequality dimension showed that women with primary education had the most upward increased use of modern contraceptives in 2003 and 2018, with 11.18% and 14.07% in both surveys, respectively. Women residing in the urban area in Nigeria dominated modern contraceptive use in both surveys, with 13.94% (2003) and 18.21% (2018). A sub-national region in Nigeria had a decline in modern contraceptive use among women of reproductive age in the South East region with an inequality margin of -0.15 between 2003 (13.01) and 2018 (12.86) (Table 1).

**Table 1:** Trends in modern contraceptive use inequality among women of reproductive age in Nigeria between 2003 and 2018

Dimensions	2003 (8.25%) *N* = 5,336		2018 (12.01%) *N* = 29,090	
	Sample (*n*)	% (95% CI)	Sample (*n*)	% (95% CI)
**Age group**				
15–19	544	3.85 (1.82–7.97)	1927	2.31 (1.67–3.18)
20–49	4791	8.75 (7.81–9.79)	27,163	12.70 (12.01–13.42)
**Economic status**				
Quintile 1 (Poorest)	1150	3.58 (2.43–5.26)	6008	3.65 (3.06–4.36)
Quintile 2	1142	2.87 (1.87–4.38)	6224	6.25 (5.12–7.61)
Quintile 3	1086	6.70 (5.2–8.58)	5601	11.16 (10.13–12.28)
Quintile 4	957	9.18 (7.11–11.76)	5599	17.90 (16.53–19.36)
Quintile 5 (Richest)	1002	20.53 (17.26–24.23)	5657	22.23 (20.76–23.77)
**Level of education**				
No formal education	2877	2.3 (1.74–3.02)	12,955	4.28 (3.78–4.84)
Primary school education	1175	11.18 (8.88-14.00)	4580	14.07 (12.79–14.47)
Secondary/higher education	1284	18.90 (16.52–21.52)	11,554	19.86 (18.85–20.91)
**Place of residence**				
Rural	3703	5.74 (4.87–6.74)	17,299	7.78 (7.09–8.55)
Urban	1633	13.94 (12.06–16.07)	11,790	18.21 (17.21–19.35)
**Sub-national region**				
North Central	754	10.33 (8.22–12.91)	4086	13.72 (12.30-15.26)
North East	1122	3.05 (2.3–4.03)	4841	7.84 (6.83–8.98)
North West	1880	3.31 (2.49–4.38)	9826	6.24 (5.11–7.59)
South East	368	13.01 (9.62–17.36)	2893	12.86 (11.42–14.45)
South South	664	13.80 (10.20–18.40)	2777	15.69 (14.04–17.49)
South West	548	23.08 (19.20-27.48)	4666	24.29(22.63–26.02)

### Inequality estimated indices of factors associated with modern contraceptive use among women of reproductive age in Nigeria between 2003 and 2018

Table 2 indicates the estimated indices of factors associated with modern contraceptive use among women of reproductive age in Nigeria. Simple (D and R) and complex (PAF and PAR) were the indices used to estimate inequality. The absolute inequality estimate for modern contraceptive use among women of reproductive age in Nigeria for all the dimensions included indicators increased from 2003 to 2018 except for educational level and sub-national regions. Furthermore, all estimated factors showed a mild inequality gap in modern contraceptive use among women of reproductive age in Nigeria, except for the age of women.

The simple measures showed a substantial inequality gap in the age of women in Nigeria (*R* = 2.27 and 5.51) in the 2003 and 2018 surveys, respectively. Other factors, such as economic status, showed mild inequality because R’s estimated inequality gap between 2003 and 2018 was 1.63 and 0.35, respectively. A marginal inequality gap was observed in the sub-national region in modern contraceptive use among women of reproductive age in Nigeria, with *R* = 7.58 in 2003 and *R* = 3.89 in 2018. Concerning complex measures, the level of education showed a PAF of 129.11 and 65.39 in 2003 and 2018, respectively (Table 2).

**Table 2:** inequality estimated indices of factors associated with modern contraceptive use among women of reproductive age in Nigeria between 2003 and 2018

Dimensions	2003			2018		
	Estimate	Lower bound	Upper bound	Estimate	Lower bound	Upper bound
**Age**						
Difference (D)	4.90	1.90	7.90	10.39	9.37	11.42
Population Attributable Fraction (PAF)	6.06	-13.57	25.69	5.73	0.11	11.35
Population Attributable Risk (PAR)	0.50	-1.12	2.12	0.69	0.01	1.36
Ratio (R)	2.27	1.08	4.79	5.51	3.97	7.65
**Economic status**						
Difference (D)	16.94	13.21	20.67	18.57	16.94	20.21
Population Attributable Fraction (PAF)	148.85	136.31	161.39	85.09	81.26	88.91
Population Attributable Risk (PAR)	12.28	11.24	13.31	10.22	9.76	10.68
Ratio (R)	5.73	3.77	8.71	6.08	5.03	7.36
**Level of education**						
Difference (D)	16.60	14.04	19.16	15.59	14.43	16.74
Population Attributable Fraction (PAF)	129.11	122.98	135.24	65.39	62.75	68.03
Population Attributable Risk (PAR)	10.65	10.14	11.15	7.85	7.54	8.17
Ratio (R)	8.23	6.07	11.14	4.65	4.06	5.31
**Place of residence**						
Difference (D)	8.21	6.01	10.40	10.42	9.09	11.76
Population Attributable Fraction (PAF)	69.05	62.59	75.51	51.62	49.05	54.19
Population Attributable Risk (PAR)	5.7	5.16	6.23	6.20	5.89	6.51
Ratio (R)	2.43	1.96	3.02	2.34	2.09	2.62
**Sub-national region**						
Difference (D)	20.03	15.83	24.23	18.05	15.96	20.14
Population Attributable Fraction (PAF)	179.82	167.95	191.70	102.21	98.62	105.80
Population Attributable Risk (PAR)	14.83	13.85	15.81	12.28	11.84	12.71
Ratio (R)	7.58	5.44	10.55	3.89	3.16	4.80

## Discussion

The present study assessed the inequality gap in modern contraceptive use among women of reproductive age in Nigeria between 2003 and 2018. The study showed an upward increase in modern contraceptive use among women of reproductive age in Nigeria. Similarly, the study reported an inequality gap in Nigerian women of reproductive age, wealth classification (quintile), educational attainments, place of residence and sub-national region of residence in modern contraceptive use for both surveys. Additionally, the absolute inequality showed a mild inequality gap in modern contraceptive use among women of reproductive age in Nigeria, except for the women’s age group.

A rise in the prevalence of modern contraceptive use among women of reproductive age in Nigeria from 8.25% in 2003 to 12.01% in 2018, given an upward increase of 3.76% in modern contraceptive use among women of reproductive age in Nigeria in the study. The prevalence rate of modern contraceptive use among Nigerian women prevalence for 2018 (12.01%) reported by the study is higher than the prevalence of modern contraceptive use (10.3%) reported by Johnson [36] in another study previously conducted in Nigeria. However, another study conducted in Nigeria by Alo et al. [37] reported modern contraceptive use prevalence rate was 17.2%, which is higher than the prevalence reported in this study for both 2003 (8.25%) and 2018 (12.01%).

The difference in the prevalence rate of modern contraceptive use among women of reproductive age in Nigeria, as reported by Johnson [36] and Alo et al. [37], and this study could be attributed to the data source of both studies. Whilst Johnson [36] utilised the 2013 NDHS dataset, Alo et al. [37] utilised the performance monitoring accountability (PMA) dataset. Despite the variations in the reported prevalence of modern contraceptive use among women of reproductive age in Nigeria, there is still evidence of low modern contraceptive use compared to other counterpart Africa countries. For instance, studies conducted in Ghana reported a prevalence rate of modern contraceptive use of 21% [38] and 21.5% [39]; in the same vein, another study conducted by Shagaro et al. [40] in Ethiopia reported a 38.7% prevalence whilst a study conducted by Kalinda et al. [41] in Rwanda reported a 52.4%.

Furthermore, the use of modern contraceptives was more pronounced among women of reproductive age between the age of 20–49 in both surveys, with corresponding 8.75% in 2003 to 12.70% in 2018 compared to women between the age of 15–19. Inequality in the age group access to sexual and reproductive health services, including modern contraceptives in developing countries, including Nigeria, continues to be a major challenge despite the robust campaigns and policies to accelerate adolescent girls’ access to safe and affordable modern contraceptives in Nigeria [42–44]. Similar age-related inequalities in modern contraceptive use were reported in a study conducted in Indonesia by Yusuf et al. [38] and another study by Nyarko [45] in Ghana.

Regarding the economic status of women, Nigerian women within the quintile 5 (richest) dominated the progressive use of modern contraceptives from 20.53 to 22.23% in 2003 and 2018, respectively. Women’s socioeconomic strength is often associated with the ability to access healthcare commodities, including modern contraceptives [26, 46–48]. In Africa, where the poverty rate is high, the opportunity cost on the choice of commodities to use the money on becomes imperative [49, 50], as women in Nigeria within the lower quintile may find it difficult to be able to forgo other life-sustaining commodities for modern contraceptives. The result of this study corroborates with a study conducted by Andi et al. [51] in Uganda, which concluded that women between the second to fifth wealth quintile were more likely to use modern contraceptives compared to their counterparts within the lowest quintile.

An upward increase in modern contraceptive use among women of reproductive age in Nigeria with primary educational attainment was reported in this study. Educational attainment has been an important medium in enlightening women’s health needs, which, in turn, accelerates the use of healthcare services [52]. Studies have reported that women’s health autonomy is directly linked to educational level, which can influence women’s choice to make an informed decision regarding modern contraceptive use [39, 51].

Finally, this current study showed that women in rural areas were less likely to use modern contraceptives in Nigeria; similarly, a downward prevalence in modern contraceptive use was reported in a region (South East region) in Nigeria. Regional and residential variation in modern contraceptive use has been noted as a major obstacle in family planning use [26, 45], particularly in Africa as a continent [53]. As noted by Tariq et al. [47], one of the challenges could be associated with logistics in the distribution of family planning commodities. Access to remote and non-motorable areas could affect the stocking and re-stocking of women’s choice of modern contraceptives [54, 55]. Inequalities in place and region of residence could impede envisaged progress in achieving universal sexual and reproductive health coverage, including SDGs targets 3.7 and 5.6 [56, 57].

### Implications for policy and public health practice

This study’s results on age, wealth quintile, educational level, and residential and regional inequality gaps in modern contraceptive use among women of reproductive age in Nigeria emphasised the need to consider public health strategies to develop policies to reduce the inequality gap in modern contraceptive use among women of reproductive age in Nigeria. The envisaged interventions or policies to bridge the inequality gap in modern contraceptive use among women of reproductive age in Nigeria should consider including pro-adolescent components and improving distribution access to women in remote and rural areas.

### Strengths and limitations

This current study’s strengths lie in using reliable and robust WHO HEAT software to analyse the inequality gap in modern contraceptive use among women of reproductive age in Nigeria. The platform allowed the use of both simple and complex measures of inequality, which gives a clear understanding of the inequality dimensions included in this study. Another strength of the study is the use of the NDHS dataset, which is a nationally representative dataset which enables the study’s findings to be generalisable to the Nigerian women population. However, the study’s limitation is that the study didn’t include some variables, such as health insurance coverage, which are important in determining equity in modern contraceptive use. This study dataset is from a cross-sectional survey, which limits the establishment of causality. Finally, due to the automated categorisation of the variables included in this study on the WHO HEAT software application page, the author is unable to recategorise or add other important variables, such as marital status in this study.

## Conclusion

The study concluded that modern contraceptives among women of reproductive age in Nigeria between 2003 and 2018 showed an increase in the prevalence rate of modern contraceptive use from 8.25 to 12.01%. However, the prevalence rate is still low compared to other African countries. The study also identified an inequality gap in the use of modern contraceptives based on age, wealth quintile, educational attainment, place of residence, and sub-national region of residence. Modern contraceptive use was more pronounced among women of reproductive age 20–49 and those within the wealthiest quintile. Women with primary educational attainment were also more likely to use modern contraceptives. Rural residents and certain sub-national regions were associated with a lower prevalence rate of modern contraceptive use. The study highlights the need for policies and programs that target the groups with low use of modern contraceptives to promote equity in family planning services.

## Data Availability

The datasets utilised in this study can be accessed at https://dhsprogram.com/data/available-datasets.cfm.

## Abbreviations

D: Difference
R: Ratio
PAR: Population attributable risk
PAF: Population attributable fraction
LMICs: Low-and middle-income countries
SSA: Sub-Saharan Africa
UNFPA: United Nations Population Fund
NDHS: Nigeria Demographic Health Survey
HEAT: Health Equality Assessment Toolkit
WHO: World Health Organisation
DHS: Demographic Health Survey
EAs: Enumeration areas
CI: Confidence interval
PMA: Performance monitoring accountability
DHS: Demographic Health Survey
SDGs: Sustainable Development Goals
